# Brain endothelial cell‐targeted gene therapy of neurovascular disorders

**DOI:** 10.15252/emmm.201606407

**Published:** 2016-05-10

**Authors:** Serena Marchiò, Richard L Sidman, Wadih Arap, Renata Pasqualini

**Affiliations:** ^1^ University of New Mexico Comprehensive Cancer CenterAlbuquerqueNMUSA; ^2^ Division of Molecular MedicineDepartment of Internal MedicineUniversity of New Mexico School of MedicineAlbuquerqueNMUSA; ^3^ Department of OncologyUniversity of TurinCandioloItaly; ^4^ Candiolo Cancer Institute‐FPOIRCCSCandioloItaly; ^5^ Department of NeurologyBeth Israel Deaconess Medical CenterHarvard Medical SchoolBostonMAUSA; ^6^ Division of Hematology and OncologyDepartment of Internal MedicineUniversity of New Mexico School of MedicineAlbuquerqueNMUSA

**Keywords:** Cardiovascular System, Genetics, Gene Therapy & Genetic Disease, Neuroscience

## Abstract

Neurovascular disorders are difficult to treat due to the blood–brain barrier (BBB), which prevents delivery of most drugs from the blood into the brain. Gene therapy can potentially overcome this barrier, but classical vectors are neither efficient nor specific enough to provide long‐lasting treatment or avoid off‐target effects. In this issue of *EMBO Molecular Medicine*, Körbelin *et al* (2016) describe an engineered adeno‐associated virus (AAV) with exceptional tropism for the brain that restores a normal phenotype in a mouse model of *incontinentia pigmenti* (IP), a severe human genetic disorder of brain vasculature.

Neurovascular disorders are a heterogeneous group of pathologies that display a common characteristic: they are difficult to treat with traditional pharmacological approaches. This is largely due to restraints imposed by the BBB that limit drug distribution to neurons and glial cells of the central nervous system (CNS) upon systemic administration. The BBB is a highly selective, active interface composed mainly of vascular endothelial cells, but also astrocytes and pericytes, as well as neurons, microglial cells, and smooth muscle cells. In the brain and spinal cord, endothelial cells lining the entire inner surface of blood vessels are linked to one another by tight junctions, which inhibit transport of most compounds across the BBB. Astrocyte glial cells envelop the outer surface of small blood vessels in the CNS and impose further restrictions.

In this issue of *EMBO Molecular Medicine*, Körbelin *et al* (2016) apply an innovative *in vivo* screening system to isolate engineered AAVs that can deliver transgenes specifically to the brain in mouse models. The authors exploited an AAV2 library displaying random 7‐amino acid insertions on the capsid protein, which was injected intravenously for reiterated affinity selection for brain‐homing vectors. An AAV displaying the NRGTEWD peptide (named AAV‐BR1) was identified that showed high specificity accompanied by high transgene expression in the brain with minimal off‐target affinity (including for the liver, for which the wild‐type AAV2 exhibits a strong tropism). AAV‐BR1 revealed substantially improved transduction efficacy over AAV‐PPS, an AAV2 vector functionalized with a phage display‐selected, brain‐specific peptide (Chen *et al*, 2009). Stable and specific transgene expression was detected *in vivo* for > 660 days by luciferase‐mediated bioluminescence. This was confirmed *ex vivo* with a relative brain/liver luciferase activity of 1,000 for AAV‐BR1 compared to 10 for AAV‐PPS (which was predominantly detected in the heart).

Although most AAV‐BR1, like other AAVs, was non‐specifically entrapped by the reticuloendothelial system in the spleen, this organ was not transduced, demonstrating that ligand‐directed AAV delivery is required for full functionality. In addition, brain homing was also accompanied by detargeting of other sensitive sites, for example, liver, heart, and kidneys. The authors identified brain vessel endothelial cells as the main target of AAV‐BR1, which broadly transduced CNS‐associated capillaries in various tested brain areas (cerebellum, olfactory bulb, striatum, cerebral cortex), as well as in the spinal cord. They also observed sporadic neuronal transduction, demonstrating that AAV‐BR1—at least to some extent—can cross the BBB, possibly by transcytosis through the endothelial layer.

As a proof‐of‐concept toward medical applications, the authors used a murine model of IP (a severe human neurovascular disease) to evaluate the therapeutic effects of AAV‐BR1 (Fig 1). IP is caused by heterozygous mutations that inactivate the *Nemo* gene (Smahi *et al*, 2000); patients exhibit a mild dermal phenotype associated with major neurological symptoms and epilepsy due to loss of brain capillaries and disruption of the BBB (Meuwissen & Mancini, 2012). Injection of a single intravenous dose of the AAV‐BR1 vector carrying a wild‐type *Nemo* gene (AAV‐BR1‐NEMO) completely reversed the neurovascular abnormalities in both heterozygous (Nemo^−/+^) and brain endothelial cell‐conditionally null (Nemo^beKO^) mouse models of IP. Besides its efficacy at the diseased sites, two aspects of this ligand‐directed gene therapy are noteworthy in terms of clinical safety: (i) Overexpression of *Nemo* did not affect BBB tightness in wild‐type animals, and (ii) AAV‐BR1‐NEMO did not transduce peripheral endothelial cells in either normal or diseased animals. The latter is of particular relevance because *Nemo* upregulation in non‐CNS blood vessels can lead to severe adverse effects such as atherosclerosis (Gareus *et al*, 2008).

**Figure 1:**
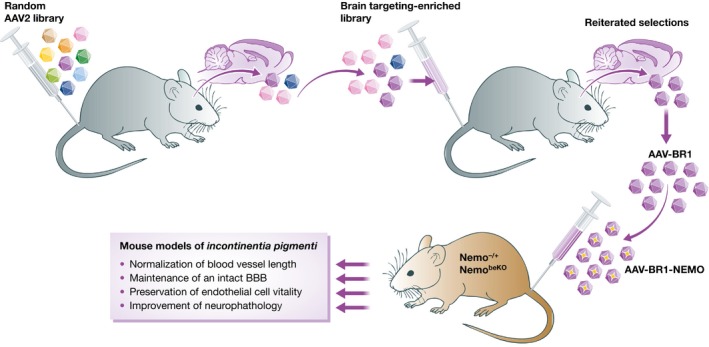
Selection and functional validation of brain‐targeting AAVs Libraries of AAV2 vectors expressing random peptide inserts on their capsid proteins were injected intravenously in mice. The population of AAVs enriched in the brain was subjected to reiterated selection rounds, leading to the identification of a candidate lead viral vector (AAV‐BR1), which was exploited to selectively deliver a therapeutic transgene (AAV‐BR1‐NEMO) to mouse models of *incontinentia pigmenti*, a severe human genetic disorder of brain vasculature.

The data from Körbelin *et al* (2016) are a promising step toward more effective therapeutic approaches targeting the CNS, where synergistic effects can be achieved by a concomitant intervention on blood vessels and, potentially, neurons. Both cell types, indeed, could be transduced *in vivo* by AAV‐BR1. So far, specific brain transduction has only been observed with AAVs injected locally and at high doses (de Backer *et al*, 2010; Markakis *et al*, 2010). Although some natural, for example, AAV2 and AAV9, and surface‐modified serotypes can cross the BBB (Foust *et al*, 2009; Yang *et al*, 2014; Choudhury *et al*, 2016), their systemic administration results in substantial targeting of non‐CNS tissues such as heart, liver, and skeletal muscle (Zincarelli *et al*, 2008). The only AAV vector described to transduce brain—but also cardiac—microvascular cells is the previously mentioned AAV‐PPS (Chen *et al*, 2009). However, Körbelin *et al* (2016) elegantly demonstrate enhanced functionality of AAV‐BR1, identifying it as the first efficient and specific vector for *in vivo* systemic administration of a transgene to the brain endothelium.

Brain endothelial cells not only contribute to the BBB: as a key component of cerebral blood vessels, they are functionally involved in a plethora of neurological pathologies including stroke, multiple sclerosis, epilepsy, and cancer. Depending on the delivered transgene, transduction of CNS endothelial cells with the AAV‐BR1 vector could potentially be tuned to improve cerebral perfusion after a stroke, impair vascularization of brain tumors, repair or otherwise modulate the BBB, and directly target the CNS through systemic blood circulation. However, although genetically stable, endothelial cells reveal an exceptional phenotypic plasticity in pathologic conditions, a well‐described scenario, for example, in cancer. This implies remodeling of their surface proteome, leading to differential receptor exposure and/or accessibility. As a consequence, a targeting ligand selected in normal conditions, such as the peptide motif described by Körbelin *et al* (2016), might not be broadly applicable, and selection of disease‐specific addresses will probably be necessary to achieve similar results in different contexts. Moreover, for efficient transgene delivery such a ligand‐targeted AAV must not only recognize its specific receptor on the cell surface but also undergo active (receptor‐mediated) internalization. Both events may impact on signal transduction, with different outcomes depending on the receptor's function(s). Interference of gene therapy‐induced intracellular pathways with the desired transgene effect is not expected to be an issue when targeting a suicide gene to cancer tissues, but should be carefully evaluated in the case of therapeutic genes. In this regard, the efficacy of AAV‐BR1‐NEMO in the IP mouse model represents an encouraging result in light of future applications.

In conclusion, Körbelin *et al* (2016) provide a pioneering work to address very relevant and unmet clinical needs that go beyond AAV as a gene therapy vector for the proof‐of‐concept genetic disorder investigated. AAV display peptide libraries can be selected to target any organ of interest, providing a valuable strategy for applications in many other diseases not associated with the neurovascular system. AAVs are recognized for their low immunogenicity, ability to transduce non‐dividing cells, long‐lasting transgene expression, non‐pathogenicity, and clinical safety. Surface‐engineered vectors, such as the one discovered here, add another key clinically relevant characteristic: unprecedented specificity.
